# Renal Impairments in Stone Quarry Workers

**DOI:** 10.7759/cureus.48743

**Published:** 2023-11-13

**Authors:** R Bama, Manikandan Sundaramahalingam, Nag Anand

**Affiliations:** 1 Physiology, Bharath Institute of Higher Education and Research, Bharath University, Chennai, IND; 2 Physiology, Indira Medical College and Hospitals, Chennai, IND; 3 Physiology, Tagore Medical College and Hospital, Chennai, IND; 4 General Medicine, Sree Balaji Medical College and Hospital, Chennai, IND; 5 General Medicine, Bharath Institute of Higher Education and Research, Bharath University, Chennai, IND

**Keywords:** quarry workers, kidney damage, heavy metal toxicity, bio markers in acute kidney injury, nephrotoxicity, health related air pollution, occupational health hazards

## Abstract

Introduction

Exposure to dust due to stone quarrying can cause severe respiratory ailments. Besides lung problems, research shows that exposure to quarry dust may also increase the risk of health problems affecting the heart, liver, kidney, central nervous system, and other organs. Despite the fact that a lot of studies have been reported on the respiratory system, our aim was to explore the evidence on the association between occupational exposure to quarry dust and its effect on renal health.

Methodology

This study was conducted on 75 quarry workers and 45 healthy matched controls were recruited from Allukuzhi village. Blood samples were collected and their kidney parameters were assessed in Hitech Diagnostics, Kanchipuram. Data were analyzed using ANOVA and strength of association was determined by Pearson correlation at significance p=0.05*.

Results

The obtained results showed a significant increase in the level of creatinine (1.02±0.31), urea (24.62±8.52), and uric acid (5.13±1.31) in quarry workers upon the duration of exposure to quarry dust compared with control subjects (p < 0.05*).

Conclusion

The results of this study suggest a significant correlation between exposure to quarry dust and its reduced renal function. This could suggest that the quarry work site should have proper hazard control measures and safety precautionary equipment for the workers. Also, to be educated about the importance of the safety measures which have to be practiced in order to protect them from occupational hazards.

## Introduction

Quarrying is the process by which rock, sand, gravel, or other minerals from the earth are removed to produce materials for construction work and other uses. Although it can cause serious medical conditions, quarrying plays an important role in the lives of rural people in developing countries because it provides their livelihood needs. Stone quarrying involves a collective process by which rock is extracted from the earth’s crust and crushed to produce aggregate sizes [1]. It is a process by which heavy metals, organic solvents, and silica are excavated from the earth by blasting, drilling, and crushing. Workers exposed to such an environment include miners, quarry workers, ceramic workers, glass manufacturers, and masons. During the process of quarrying, large amounts of dust particles of heavy metals, silica, and organic solvents are emitted from the earth's surface. Isara [2] has reported a lack of awareness of hazards associated with work and the use of safety equipment among quarry workers in quarry industries. Therefore, inhalation of this dust from the quarry can result in heavy metal toxicity causing damage to the central nervous system, heart, lungs, kidneys, liver, or other organs. The dust from quarries containing silica, heavy metals, or organic solvents enters the human body through water, food, air, or skin. Acute poisoning occurs due to inhalation or skin contact with vapors, fumes, or dust.

Heavy metals are natural elements of the earth’s crust and are released into the environment through factories, industries, municipal waste management, transport, and fertilizers [3]. Granite in the Ebony State of Nigeria has a high concentration of silicon dioxide, along with contents like aluminum oxide, lead, mercury, and arsenic [4]. The emission of heavy metals occurs frequently in air, water, and soil and has significant toxicity In vivo and in vitro studies were reported by Markiewicz-Gorka [5]. High bioaccumulation and mobility were noticed in Cadmium in living Organisms [6]. The emission of heavy metals causes major health hazards because they are resistant to decomposition and because of bioaccumulation and biomagnification [7,8]. The dust from quarries is a trait of systemic and visceral organs [9,10] affecting the safety and health of quarry workers and the people around them. This can give rise to serious health threats like silicosis, cardiovascular, liver, and kidney problems [11]. These diseases are because of the induction of some reactive oxygen species (ROS) by silicon dioxide, aluminum oxide, and heavy metals like lead, cadmium, mercury, chromium, and platinum. Heavy metals, which can be toxic with very low doses and are non-biodegradable with long biological half-life, can accumulate in the kidneys where they can cause various kinds of nephropathy [12], in addition to hypertension, neurodegenerative diseases, and cognitive impairment [13]. Silica can be directly toxic on the glomerulus and proximal tubule which is a possible mechanism to cause renal damage. Saldhana and Haustein [14] have proposed an immune pathway that was possible for silica-induced scleroderma and may be a relevant mechanism for other autoimmune conditions, which include end-stage renal disease.

Various studies have been reported on quarry workers and their renal health. The kidney is a target organ in heavy metal toxicity for its potential to reabsorb and concentrate divalent ions and metals [15]. Kidney impairment depends on the nature, dose, and duration of exposure. Studies have reported that heavy metal damages the kidney by accumulating in the renal cells and by blocking metabolic processes, initially in the tubule, leading to proteinuria and necrosis. The aim of this study is to investigate the impact of quarry dust on the kidney health of quarry workers, with the objective of reinforcing the evidence base for necessary health and safety precautions.

## Materials and methods

Study area

The study was conducted in a village called Yelaanjeri, Vempakkam Vattam which is located in Thiruvannamalai district, 120 km away from Chennai, the Capital of Tamil Nadu, India. It is a small village having numerous quarries being flanged on all sides of the roads and almost the entire population depends on quarry industries for their living. Likewise, the control subjects were recruited from an area called Allikuzhi village, Thiruvallur district, Tamil Nadu, India. The prior informed consent was obtained from the Quarry industry and all the participants with ethical clearance obtained from the Institutional Ethical Committee (IEC/Jan/19), Tagore Medical College and Hospital, Chennai, Tamil Nadu, India.

Participants

The people of the village are inhabited by mostly quarry workers as the entire village is completely surrounded by hard rock areas with very little agricultural land. Hence, the entire population depends predominantly on quarries for their living. Our subjects included 120 aged between 20 and 55 years among these 75 were worked for two to 25 years in quarry industries and 45 participants were age-matched control subjects who were healthy people. The inhabitants of this village had no exposure to quarry dust as there were no quarries in and around 20 km.

Preliminary data were collected by way of face-to-face interviews and well-structured questionnaires, to determine the duration of exposure, date of employment, site or position at the workplace, use of personal protective equipment such as dust masks and earplugs, etc. Information on general health, history of past diseases, and habits such as smoking and alcohol consumption were also obtained.

Inclusion criteria

Males between the age group of 20 and 55 working in the quarry for more than two years and are willing to participate are included in the study.

Exclusion criteria

Subjects who had not participated in stone quarrying for less than two years and those who were not doing it as a full-time job were excluded from the study. This was to ensure that there was constant and sustained exposure to quarry dust. Workers with clinical abnormalities of the vertebral column and thoracic cage, hemoptysis, pulmonary tuberculosis, chronic bronchitis, emphysema, and other respiratory diseases, anemia, diabetes mellitus, secondary hypertension, jaundice, kidney diseases, and subjects who had undergone chest or abdominal surgery were excluded from the study.

Sample collection

Blood samples were collected from the site from midmorning to noon for both the subjects and controls. Prior consent was taken from all the subjects by reading and explaining the purpose of the work. 5mL of blood sample was collected by vein puncture under aseptic conditions into a dry, clean heparinized tubes for hematological assays. 3mL of blood sample was left to coagulate and was centrifuged at 3,500 rpm for five minutes. After centrifuging, the serum was separated and dispensed into a dry clean serum container, after which the samples were analyzed immediately and stored at -20°.

Biochemical assays

Serum creatinine was done by (Jaffe’s method) Urea by (GLDH/urease method) and uric acid by (Uricase /POD method) and were assayed at hi-tech diagnostic laboratories, Kanchipuram using reagent kits purchased from (Autospan), and also were carried out at Department of Biochemistry, Indira Medical College and Hospital, Thiruvallur. 

Statistical analysis

Data were analyzed using the SPSS 21.0 version (IBM Corp., Armonk, NY) and expressed as mean ± standard deviation (SD). All statistical comparisons were performed using a one-way ANOVA analysis of variance, and unpaired “t” test for comparison between quarry workers and controls. Correlations were used to test for linear relations between quantitative variables using Pearson’s correlation analysis. Univariate analysis tested for significant predictors of kidney function and duration of exposure. P<0.05* or less was considered for statistical significance.

## Results

**Figure 1:**
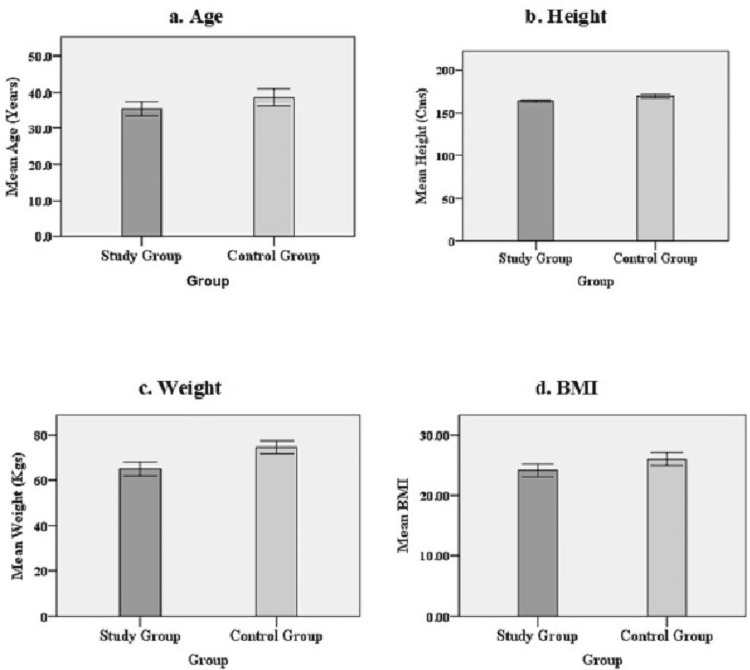
Demographic features for the study group (n=75) and control group (n=45). Data represent the difference between quarry and control subjects of (a-d) age, height, weight and BMI.

A total of 120 participants (75 quarry workers and 45 controls) were enrolled in the study, with a median age of 37 (interquartile range: 20) years. The matched control individuals selected from the village site were such that they were age and gender-matched with that of the quarry workers. Demographic data for the study population are presented in Figures 1a-1d of exposed and control groups matched in age, height, weight, and BMI.

Tables 1, 2 show duration of exposure among which the majority of quarry workers 36 (48%) had worked for a period of less than 5 years (𝑝=0.001*), 29 (38.7%) had worked for 5-10 years (𝑝=0.001*), and 10 (13.3%) had worked for more than 10 years (𝑝=0.001*) which shows significant changes in the duration of exposure.

**Table 1:** Exposure and years of exposure for the study group and control group. Data presented are duration of exposure on Quarry workers compared to control subjects.

Exposure	Years of Exposure	Study Group (n=75)		Control Group (n=45)	
		n	%	N	%
Yes	≤ 5 years	36	48.0%	0	0.0%
	5-10 years	29	38.7%	0	0.0%
	≥ 10 years	10	13.3%	0	0.0%
Nil		0	0.0%	45	100.0%
Total		75	100.0%	45	100.0%

**Table 2:** Univariate ANOVA test - differences in biochemical parameters of kidney by year of exposure to quarry (n=75). Data presented are mean±SD. Analysis of data was done by one-way ANOVA and Student “t” test to compare Quarry subjects with Control participants. The significant difference (P<0.05*) is flagged with * and (P<0.001) is **.

Parameters	Mean (Std. Deviation) for year exposure to quarry (n=75)					
	≤ 5 years (n=36)		5-10 years (n=29)		≥ 10 years (n=10)	
Urea	22.13	(4.89)	30.62	(4.67)	40.22	(2.33)
Creatinine	0.86	(0.10)	1.02	(0.13)	1.41	(0.33)
Uric Acid	4.64	(0.77)	6.64	(0.63)	7.34	(0.49)
Source		Sum of Squares	Df	Mean Squares	F	P-value
Urea						
Between Groups		2929.82	2	1464.91	70.49	.00**
Within Groups		1496.23	72	20.78		
Total		4426.06	74			
Creatinine						
Between Groups		2.40	2	1.20	49.36	.00**
Within Groups		1.75	72	0.02		
Total		4.15	74			
Uric Acid						
Between Groups		92.55	2	46.28	97.20	.00**
Within Groups		34.28	72	0.48		
Total		126.83	74			

Table 3 shows the mean differences in kidney parameters and levels of creatinine were significantly higher in the quarry workers (1.02±0.24) than the control samples (0.77±0.24) p < 0.05*, which showed the difference and the level of urea in quarry workers were markedly higher (27.82±7.73) compared to control sample (18.72± 3.25) p < 0.05*. Likewise, the level of uric acid was significantly higher in quarry workers (5.77±1.31) than the control subjects (3.76± 0.87) p < 0.05*.

**Table 3:** Independent “t” test - differences in biochemical parameters of kidney between study group and control group. Data presented are means±SD. The independent “t” test analysis done for kidney parameters to compare Quarry workers with Control participants. The significant difference (P<0.05*) is flagged with * and (P<0.001) is **.

Variables	Group	n	Mean	Std. Deviation	t	df	P-value
Urea	Study Group	75	27.82	7.73	7.50	118	.00**
	Control Group	45	18.72	3.25			
Creatinine	Study Group	75	1.00	0.24	5.08	118	.00**
	Control Group	45	0.77	0.24			
Uric acid	Study Group	75	5.77	1.31	9.16	118	.00**
	Control Group	45	1.76	0.87			

Table 4 shows the correlation analysis to quarry dust against the kidney function in study group. A significant positive correlation (𝑟= 0.38, 𝑝 = 0.001*) was observed in the exposed subject than control group.

**Table 4:** Pearson correlation analysis of kidney parameters in study group and control group. Data represent the correlation levels of kidney parameters in Quarry and Control group to determine the strength among the variables. **Correlation is significant at the level 0.01. *Correlation is significant at the level 0.05.

Variables	Study Group (n=75)		Control Group (n=45)	
	r value	P value	r value	P value
Urea	.38	.00**	.10	.52
Creatinine	.20	.04*	.20	.18
Uric acid	.38	.00**	.10	.50

## Discussion

The aim of this study was to investigate the impact of inhalation of quarry dust on renal function, by analyzing the biochemical changes with respect to duration of exposure to dust in quarry workers. Based on the inclusion and exclusion criteria, 75 male quarry workers, involved in blasting, crushing, and drilling for more than two years were recruited for the study. Age and gender-matched 45 non-exposed subjects were recruited from the area where no quarries were surrounded for about 20 km for this study.

The Polluted air containing heavy metals, silica, and organic solvents enters the body by inhalation and causes potential damage to the lungs. The damage caused is directly related to the concentration of the dust in the air and the duration of exposure [10]. These dust particle gets deposited on the respiratory tract lined by ciliated and mucus-secreting cells, which will be later swept into the gastrointestinal tract direct ingestion is also possible like in the hands, in the food area, and facial hair. In the GIT it will either damage or will get dissolved, absorbed, and then will be carried to the other organs. If the particulate size is soluble enough, it would be directly absorbed from the airways into the blood and will be carried to the organs. Inside the system, dissolved particulates or non-dissolved particulates interact with proteins to form complexes that are of transportable forms and move materials into various organs [16]. Heavy metals in plasma exist in two different patterns: protein-bound (non-diffusible) and complex/ionized (diffusible) forms. Metals are quickly cleared from the blood and are sequestered in many tissues.

In chronic intoxication, the protein bound, conjugates with metallothionein and glutathione, gets released into the blood by the liver and kidney. Following they are reabsorbed by endocytosis in segment S1 of the proximal tubule and would lead to chronic inflammation, fibrosis, and renal failure. Learning the effects of quarry dust on quarry workers, we proposed to find out the effects and their relations to the renal function in quarry workers by determining the levels of Creatinine, urea, and uric acid with standard procedures.

The results obtained from our study indicated that all the analyzed parameters to evaluate kidney function were significantly higher in quarry workers than control values upon the duration of exposure. There are significant changes observed with respect to the duration of work between five years, 5-10 years, and above 10 years. The study clearly shows prolonged exposure to quarry dust leads to reduced pulmonary functions and other health-associated hazards, which have aggravated with exposure over time. These are prominent risk factors found to be associated with lung diseases [17]. Silica is an important component of quarry dust, exposure to it is associated with renal insufficiency with an increase in creatinine level indicating the toxic effect of quarry dust in the exposed group.

In this study, the general comparison between control and exposure shows a significant difference in Creatinine level (1.00±0.77). Similarly, exposure of above 10 years showed a highly significant increase in creatinine (1.41 + 0.77) when compared with that of control groups **P<0.01. Likewise, the group exposed to 5-10 years and less than five years of exposure shows a marked increase in the levels of creatinine (1.02, 0.86), respectively, compared with that of the control group (0.77) **P<0.01. The correlation of kidney markers with duration of exposure and age also showed a positive signiﬁcance of (r=0.20, p<0.05) in this study. Creatinine is an important parameter that signifies the kidney's health and an increase in creatinine level denotes the impairment of the kidney. Al-Otaibi et al. have reported an increase in creatinine levels in stone quarry workers subjected to heavy metals which signifies impairment of the kidney due to metal toxicity. Creatinemia also were noticed in the workers, which shows dust exposure to the kidneys [18,19].

Likewise, the level of urea in quarry workers was markedly higher compared to the control sample (27.82 ±7.73, 18.72±3.25), and a progressive increase was noticed in the duration of exposure. In our study, the exposure group above 10 years showed a highly significant increase in urea (40.22 ±18.72), and group 5- 10 years and less than five years of exposure group signify a gradual increase in the levels of urea (30.62, 22.13 ± 18.72). Ogbodo et al. [20] reported an increase in urea concentration in quarry workers than in controls. The serum urea concentration is highly influenced by factors like protein intake and tissue breakdown. Protein and tissue breakdown is a constant feature in these quarry workers whose jobs involve serious physical activity, thus the longer the duration of work increases urea concentration [21]. Urea is produced during the catabolism of protein and amino acids which, filter out of blood by the glomeruli of the kidney and partially get reabsorbed with water and increases serum urea which signifies kidney damage.

The level of uric acid was (5.77 ± 3.76) **p<0.01 in the general comparison between the quarry and control groups respectively. Chronic exposure to quarry dust above 10 years showed a highly significant increase in uric acid (7.34± 3.76) compared to the control group **p<0.01. Similar effects were also noticed in the group 5-10 years and in less than five years of exposure, showing a gradual increase in the levels of uric acid (6.64, 4.64 ± 3.76), respectively, compared with that of control groups **p<0.01. Similar effects were reported in workers with lead exposure have a higher frequency of hyperuricemia and higher serum uric acid levels than those of the control group the higher serum uric acid levels may be associated with renal impairment [22].

A study by Madu et al. [23] has reported increased levels of creatinine and urea in quarry sample rats compared to the control sample rats during the dry season than the wet season. It is more likely, that the unsettled dust from quarry during the dry season has damaged the kidneys of the rats and has increased the levels of creatinine and urea. Whereas, in comparison, the rats in the wet season have not been affected by the dust particles as the dust has been precipitated during the wet season. Therefore, this study clearly signifies the effect of quarry dust on kidneys and the same could also be true in humans.

Silica nephropathy also referred to as floorboard of kidney diseases includes tubule-interstitial disease, immune-mediated disease, chronic kidney disease, and end-stage renal disease [14,24]. Calvert et al. [25] have also indicated the association between silicon exposure and increased risk for end-stage renal disease. It is also reported that prolonged exposure to silica dust causes silica nephropathy, initially showing low urine specific gravity and later causing advanced manifestations with hypertension and proteinuria. Prolonged exposure has been reported to decrease GFR progressively [26]. The liver and kidney are major depository sites of Pb [27]. Accumulation of lead in the liver and kidney has been reported to cause a tremendous increase in the levels of liver enzymes, and creatinine also relatively causes a histopathological change, indicating both liver and kidney injuries [28].

Likewise, our study clearly shows noticeable adverse alteration in the levels of kidney markers like urea, creatinine, and uric acid in various metabolic events which is presumed to be the effect of inhalation and direct ingestion of quarry dust. This presumption is well proved when the duration of exposure to quarry dust is compared with its effect on the kidney.

Limitations of the study

The control subjects from the residents were selected randomly, whereas the quarry workers were selected by purposive sampling. Restriction in taking the workers from the quarry had limited further screening. Information related to socioeconomic status, hygiene, and housing-related factors of the workers and the residents were limited in the study and hence the effects of these factors on the presence of renal ailments have not been reported.

## Conclusions

The results of this study suggest that kidney ailments due to prolonged exposure to quarry dust can cause severe complications. Due to illiteracy, lack of job opportunities, and attractive payments given by quarry owners, quarry workers are unaware of or ignore the health hazards associated with quarry work. Therefore, the government should intervene to instruct the quarry owners to maintain proper health hazard measures, provide them with proper precautionary safety gadgets, and instruct them on the importance of their usage in order to prevent health complications.
